# The effects of zinc deficiency on homeostasis of twelve minerals and trace elements in the serum, feces, urine and liver of rats

**DOI:** 10.1186/s12986-019-0395-y

**Published:** 2019-10-30

**Authors:** Qingli Yu, Xiaohan Sun, Jiali Zhao, Lan Zhao, Yanfeng Chen, Lina Fan, Zixiang Li, Yongzhi Sun, Maoqing Wang, Fan Wang

**Affiliations:** 1 National Key Disciplines of Nutrition and Food Hygiene, Department of Nutrition and Food Hygiene, School of Public Health, Harbin Medical University, 157 Baojian Road,Nanggang District, Harbin, China; 2 Department of Pediatrics, The Second Affiliated Hospital of Harbin Medical University, Harbin Medical University, Harbin, Heilongjiang Province China; 3 Public Health Inspection and Testing Institute, Heilongjiang Provincial Center for Disease Control and Prevention, Harbin, China; 4 National Key Disciplines of Nutrition and Food Hygiene, Department of Nutrition and Food Hygiene, School of Public Health, Harbin Medical University, 157 Baojian Road, Nangang District, Harbin, China; 5 Department of Epidemiology, School of Public Health, Harbin Medical University, 157 Baojian Rd, Nangang District, Harbin, Heilongjiang Province People’s Republic of China

**Keywords:** Zinc deficiency, ICP-MS, Minerals, Trace elements, Rat

## Abstract

**Background:**

Zinc deficiency can change the concentrations of minerals and trace elements in the body. However, previous studies still had many limitations.

**Objective:**

To reveal the effects of zinc deficiency on homeostasis of 16 minerals and trace elements.

**Methods:**

Forty-five rats were divided randomly into three groups: normal zinc diet (30 mg/kg), low zinc diet (10 mg/kg), and pair-fed diet(30 mg/kg). The concentrations of 16 minerals and trace elements in serum, feces, urine, and liver were measured by inductively coupled plasma mass spectrometry. The excretion of 16 elements in urine and feces were calculated and compared.

**Results:**

Zinc-deficient rats exhibited significant changes in up to 12 minerals and trace elements. The low zinc diet induced decreased excretion of zinc and concentrations of zinc in serum, feces, urine, and liver. Zinc deficiency increased feces concentrations of Mg, Cu, Se, K, Ag, Fe and Mn; decreased the concentrations of Mg, Cu, Se, K in liver and urine, and a diminished amount of Ag was observed in serum. Decreased urinary concentrations of Zn Ca, Mg, Cu, Se, K, Na, As and Cr, suggested that zinc-deficient rats increased the 9 elements’ renal reabsorption. Decreased concentrations of Ca in liver, urine, and feces, decreased excretion in urine and feces and increased serum total Ca suggested that zinc deficiency increased the redistribution of Ca in serum or other tissues. Zinc deficiency increased excretion of Cu, Se, Fe; and decreased the excretion of other 8 elements except for Ag.

**Conclusions:**

Zinc deficiency changed the excretion, reabsorption and redistribution of 12 minerals and trace elements in rats. Our findings are the first to show that zinc deficiency alters the concentrations of Ag, Cr, and As.

**Graphical abstract:**

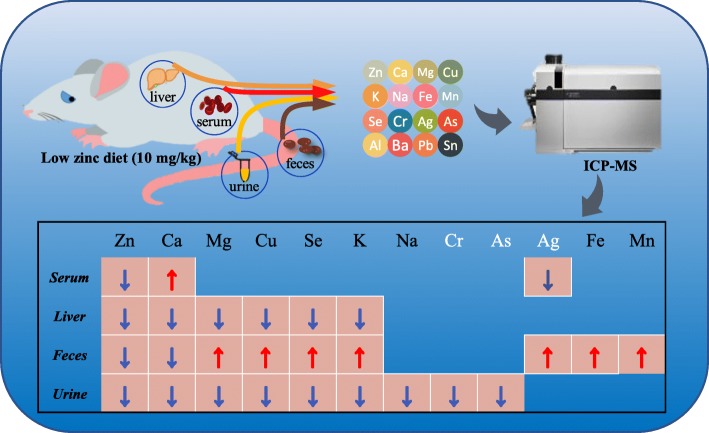

**Supplementary information:**

**Supplementary information** accompanies this paper at 10.1186/s12986-019-0395-y.

## Introduction

Zinc is an essential trace element in human body and plays extensive and important physiological functions [1, 2]. Left untreated, zinc deficiency can cause many adverse effects, including growth retardation, poor appetite, delayed sexual development, learning and memory disabilities, impaired immunity, and diabetes [1, 3–9]. Investigators have reported that zinc deficiency altered the concentrations of seven minerals and trace elements (Fe, Mn, Cu, Mg, Ca, Na, and K) in serum, urine, and tissues such as liver, bone, testes, muscle, esophagus, kidney, heart, brain, spleen, thyroid and adrenal glands, esophagus, and spleen [10–16]. In addition to zinc deficiency, abnormal concentrations of the above minerals and trace elements can also cause adverse physiological effects. For example, abnormal concentrations of iron areassociated with anemia and immunological disorders [17], manganese with nervous system diseases [18], copper with cardiovascular diseases and cancer [19, 20],calcium with BMD, magnesium and manganese with diabetes [3] and Na with hypertension. However, it is not clear whether zinc deficiency-induced alterations in the concentrations of these other elements are responsible for the foregoing adverse effects. Therefore, it is necessary to investigate the direct effects of zinc deficiency on the alterations of minerals and trace elements of body.

Existing studies about the effects of zinc deficiency on the alterations of minerals and trace elements in the body still have many limitations. First, to our knowledge, the concentrations of only two or three elements (usually Cu, Mg, and Fe) were measured in most previous studies; in only two studies researchers have measured nine or more elements simultaneously in brain tissues [14] and serum [16]. Second, the concentrations of minerals and trace elements in human or animal feces have not been reported for conditions of zinc deficiency. Likewise, there is no study of zinc-deficient animals or humans for which measurements of minerals and trace elements have been performed concurrently in serum, urine, feces, and tissues. Lastly, in most studies, investigators have used atomic absorption spectroscopy (AAS) for quantitative analysis of minerals and traces elements. Compared with AAS, inductively coupled plasma mass spectrometry (ICP-MS) can quantify multiple elements simultaneously, and ICP-MS has been applied widely for that purpose.

In this study, we used ICP-MS to simultaneously measure the concentrations of 16 minerals and trace elements in multiple samples (serum, feces, urine, and liver) of rats fed a low-zinc diet and calculate their excretion in urine and feces. By analyzing intake, excretion, and redistribution, we comprehensively revealed the effects of zinc deficiency on homeostasis of these 16 elements.

## Method and materials

### Animal experiment

The study was approved by the Harbin Medical University Institutional Animal Care Committee and performed in accordance with the Harbin Medical University guidelines for the care and use of laboratory animals. Forty-five five-week-old male Wistar rats(110-130 g) were purchased from Beijing Vital River Laboratory Animal Technology Co., Ltd. They were maintained with a controlled light schedule (12 h light: 12 h dark) at room temperature (24 ± 1 °C) and with constant humidity (50% ± 5%). After an adaptation period of 7 days, the rats were randomly divided into three groups: normal zinc diet group, *n* = 15, NZG; low zinc diet group, n = 15, LZG; and pair-fed group, n = 15, PZG. The animal diets were modified based on the standard AIN-93Gdiet with dried egg white as the protein source from Beijing KeAoLiXie Animal Food co., LTD, China. The contents of minerals and trace elements and compositions in the diets are detailed in Additional file 1: Table S1.The target content of zinc was 30 mg/kg in the normal diet and 10 mg/kg in the low zinc diet. We used flame atomic absorption spectrometry to confirm the target zinc contents; the normal diet had 30.4 mg/kg of zinc and the low zinc diet had9.7 mg/kg. The pair-fed zinc group (PZG) was fed a normal-zinc diet, and daily food intakes were same as in LZG. To avoid zinc recycling and contamination, the rats were housed singly in stainless steel cages. All rats were allowed to drink deionized water from a plastic container with a stainless-steel spray nozzle. Food intake was recorded every two days, and body weight was measured once a week.

### Sample collection, pretreatment and detection of serum biochemical indicators

#### Sample collection

Before the end of the experiment, the animals were transferred to metabolic cages with a sharp bottom funnel to collect urine and feces samples. The 24 h-urine and stools were collected twice. The volume of urine and weight of feces were recorded and the average of feces weight and urine volume was calculated and recorded. The feces of all rats were collected and placed in non-ionic EP tubes, and stored at − 80 °C.The urine of all rats was separated from feces by a sharp bottom funnel; and was collected in15ml ion-free plastic centrifuge tubes (Corning Incorporated) and subjected to centrifugation at 3000 rpm (835 g) for 10 min. The supernatants were stored at − 80 °C.After 4 weeks of feeding, all rats were fasted for 12 h and then anesthetized by intraperitoneal injection of 10% Chloral hydrate (0.3 ml/kg body weight). Blood samples were collected from abdominal aorta and placed at room temperature for 2 h. After centrifugation for 15 min at 3000 rpm(835 g), the separated serum was stored at − 80 °C. The livers were removed, washed several times with deionized water, then placed in15 ml ion-free plastic centrifuge tubes at − 80 °C.

#### Measurements of serum biochemical indicators

The concentrations of free zinc and free calcium in serum were measured by colorimetry (the reagent kits were purchased from Sigma-Aldrich, China), and the serum concentrations of metallothionein, growth hormone, interleukin (IL)-1, IL-6, thymosin, and insulin were measured by ELISA (kits from Summus, Harbin and Sigma-Aldrich).

### Quantitative analysis of16 minerals and trace elements in serum, feces, urine, and liver of rats by ICP-MS

#### Sample pretreatment

All samples were pretreated by wet digestion. Serum (200 μl) was placed in 50 ml glass conical bottles cleaned by deionization. After adding HNO_3_:HClO_4_ (4:1) solution (Guaranteed reagent, Xilong Scitenfic, China), the mixtures were digested in a temperature-controlled furnace at 100 °C until the solutions became colorless or complete. To reduce the concentration of acid in the digestive solution, 3 ml deionized water was added and dried it twice by heated. The digested materials were dissolvedwith5 ml 5% nitric acid and transferred to 15 ml ion-free plastic centrifuge tubes.

The pretreatment methods of feces, urine, and liver were the same as that for serum. Specifically, 0.05 g of feces, 200 μl of urine, and 0.1 g of liver were pretreated. The digested materials were dissolvedwith5 ml 5% nitric acid solution and transferred to a 15 ml ion-free plastic centrifuge tube.

#### Quantitative analysis of 16 minerals and trace elements by ICP-MS

The concentrations of 16 minerals and trace elements in the samples were detected with an Agilent 7800 inductively coupled plasma-mass spectrometer (ICP- MS, Agilent) in the Heilongjiang Provincial Center for Disease Control and Prevention [21]. The parameters of ICP-MS were the following: radiofrequency (RF) power: 1.55 kW; sampling depth: 8 mm; plasma gas flow: 15.0 L/min; carrier gas (Ar): 1.03 L/min; dilution gas,0 L/min; auxiliary gas, 0.9 L/min; spray chamber temperature (L): 2 °C; peristaltic pump: 0.10 r/s. Helium mode was operated in high energy mode which offers enhanced sensitivity for selenium. Helium gas flow: 10 ml/min and KED was 4 V.

A calibration standard solution (No:5183–4688) was purchased from Agilent Technology Co., Ltd. Calibration standards were prepared as follows: 10 mg/L mixed standard reserve solution was diluted with 5% nitric acid to produce mixed standard solution serials of various elements (0.5, 1, 2, 5, and 10 mg/L for K, Ca, Na, Mg, and Fe and 5, 10, 20, 50, 100 μg/L for Al, Cr, Mn, Cu, Zn, As, Se, Ag, Sn, Ba, and Pb). The standard curves were drawn with count per second (CPS) as ordinate and mass concentration as abscissa. The elemental contents in certified reference material (Trace Elements Serum L-1 RUO, Seronorm™) were determined by the established method for the determination of the elements in this study. The results confirmed the accuracy of our method.

### Statistical analysis

Serum biochemical indicators and the concentrations of minerals and trace elements were expressed as mean ± SD. Differences between the groups were analyzed by an independent t test and ANOVA test. A two-tailed value of *P* < 0.05 was considered to be statistically significant. Data were analyzed using SPSS software (version 17.0, Chicago, IL, USA).

## Results

### The weights, food intake, and serum indicators of rats

**Fig. 1:**
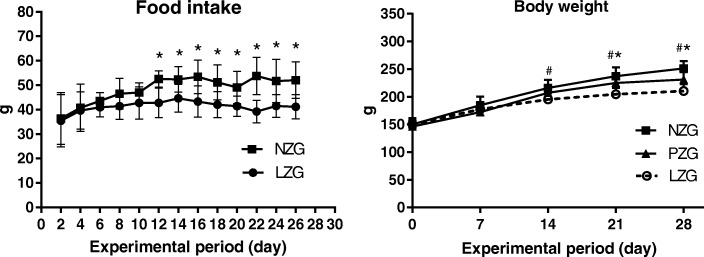
Change trends in food intakes and body weight of three groups. *: *P* < 0.05, LZG vs NZG; #: *P* < 0.05, LZG vs PZG

After 12 days of low zinc diet, the food intake of LZG was significantly lower than that of NZG (*P* < 0.01) (Fig. 1). From 21 to 28 days, the body weights of LZG were significantly lower than the weights of NZG and PZG (*P* < 0.05); and the body weights of PZG were significantly lower than the weights of NZG (*P* < 0.05) (Fig. 1).

As shown in Table 1, we observed significantly decreased serum concentrations of free zinc, metallothionein, growth hormone, interleukin (IL)-1/IL-6, thymosin, and insulin in LZG compared with NZG and PZG(*P* < 0.05). There were no significant statistical differences in all serum indicators between PZG and NZG. These findings confirmed that our zinc-deficient rat model was successfully established.

**Table 1:** Serum biochemical indicators between 3 groups

Indicators	NZG	PZG	LZG
Zinc ion (mg/L)	1.23 ± 0.25	1.10 ± 0.29	0.75 ± 0.29*
Calcium ion (mg/L)	64.72 ± 2.10	64.42 ± 3.73	65.05 ± 1.38
Metallothionein (μg/L)	6.23 ± 0.96	6.11 ± 0.89	2.05 ± 0.95*
Growth hormone (ng/L)	5.32 ± 2.06	5.43 ± 2.25	2.19 ± 1.87*
IL-1 (ng/L)	19.91 ± 6.28	18.00 ± 3.76	12.74 ± 1.69*
IL-6 (ng/L)	70.96 ± 25.90	78.60 ± 31.42	32.26 ± 10.11*
Thymosin (mg/ml)	2.89 ± 0.54	2.69 ± 0.78	0.67 ± 0.46*
Insulin (ng/ml)	0.63 ± 0.15	0.62 ± 0.17	0.42 ± 0.07*

*: *P*<0.05 LZG vs NZG and PZG

### The concentrations of 16 minerals and trace elements in the serum, feces, urine and liver of rats

#### Serum minerals and trace elements

**Fig. 2:**
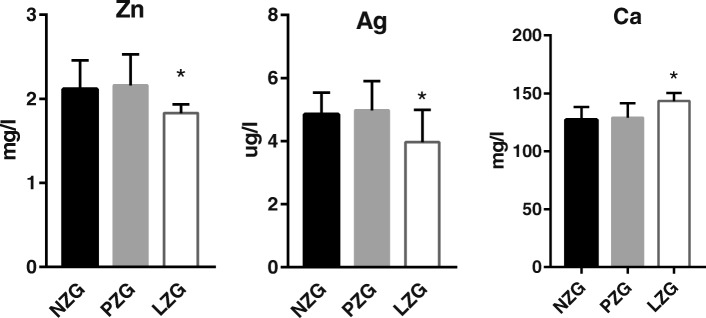
Concentrations of three minerals and trace elements in serum between three groups. *: *P*<0.05 LZG vs NZG and PZG

As shown in Fig. 2 and Additional file 1: Table S2, we found significantly decreased concentrations of total Zn and Ag in serum and significantly increased serum Ca in LZG compared with NZG and PZG (*P* < 0.05); we did not find significant differences for 13 other elements.

#### Feces minerals and trace elements

**Fig. 3:**
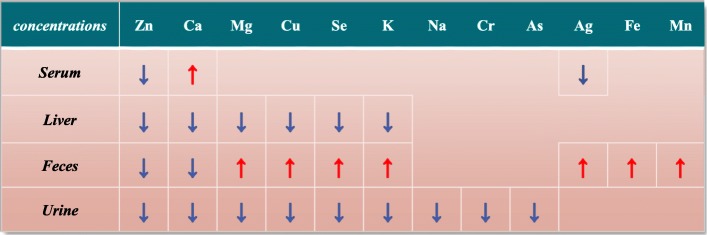
Trend analysis of concentration change of twelve minerals and trace elements in serum, feces, urine and liver between three groups. *: *P*<0.05 LZG vs NZG and PZG. The red arrow represents a significant increase: ↑,LZG vs. NZG; the blue arrow represents a significant decrease: ↓,LZG vs. NZG. Gray: first found in zinc deficient rats

As shown in Fig. 3, Additional file 1: Figure S1 and Table S3, in feces, the concentrations of Zn and Ca were significantly decreased and the concentrations of Mg, Cu, K, Se, Fe, Mn, and Ag were significantly increased in LZG compared with NZG and PZG(*P* < 0.05); significant changes for 7 other elements were not observed.

#### Urinary minerals and trace elements

As shown in Fig. 3, Additional file 1: Figure S2 and Table S4, the concentrations of Zn, Ca, Mg, Cu, Na, K, Se, Cr, and As in urine were significantly decreased in LZG compared with NZG and PZG (*P* < 0.05); we did not find significant differences for 7 other elements.

#### Liver minerals and trace elements

As shown in Fig. 3, Additional file 1: Figure S3 and Table S5, the concentrations of Zn, Ca, Mg, Cu, K, and Se in liver were significantly decreased in LZG compared with NZG and PZG (all *P* < 0.05), whereas 10 other elements showed no significant changes. The concentrations of 16 minerals and trace elements in the livers of PZG were not detected by us.

### Excretion of 12 minerals and trace elements in urine and feces

**Fig. 4:**
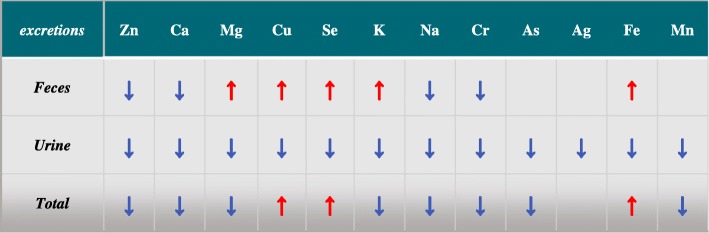
Excretions of twelve minerals and trace elements by feces and urine. The red arrow represents a significant increase: ↑,LZG vs. NZG; the blue arrow represents a significant decrease: ↓,LZG vs. NZG

As shown in Additional file 1: Figure S4, the weight of feces was significantly decreased in LZG compared with NZG and PZG (*P* < 0.05). The volume of urine in LZG suggested a decreasing trend, but the data were not statistically different between the groups. As shown in Fig. 4 and Additional file 1: Table S6, increased excretion of Mg, Cu, Se, K, Fe and decreased excretion Zn, Ca, Na, Cr were observed in the feces LZG. For reduced urine concentrations and urine volume, urinary excretion of 12 elements was all decreased in LZG or their urinary reabsorption were increased.

Figure 4 and Additional file 1: Table S7 showed that the excretion of Cu, Se and Fe in feces were greater than the reabsorption in urine. So, the total excretion of Cu, Se and Fe were increased and zinc deficient rats lost Cu, Se and Fe. In contrast, the excretion of Mg and K in feces was less than the reabsorption in urine, so the total excretion of Mg and K were decreased and zinc deficient rats did not lose Mg and K. Except for Ag, zinc deficiency increased the total excretion of Cu, Se and Fe, while the results of other 8 elements were opposite.

## Discussion

We have used ICP-MS to concurrently measure the concentrations of 16 minerals and trace elements in serum, feces, urine, and liver of zinc-deficient rats. Compared to normal diet rats, low zinc diet caused changes in the levels of 12minerals and trace elements.

### The alterations of 12 differential minerals and trace elements in four samples

Previous studies have indicated that the serum concentrations of Zn, Mn, Cu, Mg, Se, and Fe were changed by a zinc-deficient diet [11, 16]. We found decreased Zn and Ag and increased Cain serum of LZG compared with NZG (Fig. 3). The concentrations of Mn and Cu suggested a decreasing trend, but the data were not statistically different between the groups. The main reason why our test results are inconsistent with other studies were that the zinc content (≤5 mg/kg) in the low zinc diets of other studies was less than our low zinc diet(10 mg/kg), and rats were maintained on low zinc diets for longer periods than in our study. Furthermore, other investigators measured only free elements in serum, except the elements bound to proteins. Conversely, in our study, we measured the total levels of 16 elements in serum, including free elements and elements bound to serum proteins.

In liver, the concentrations of Zn, Ca, Mg, Cu, K and Se were significantly decreased in LZG compared with NZG (Fig. 3); these changes were consistent with other reports [10, 22–24]. Notably, for the first time, our results indicated that zinc deficiency caused a decrease in the concentrations of liver Se. Compared to NZG, the significantly decreased urinary Cu, Se, K, Na, As and Cr except for Zn, Mg and Ca in zinc deficient rats were first observed in our study (Fig. 3). Marginal zinc deficiency can reduce the excretion of Mg and Ca into urine and increase their retention [13],observations that are consistent with our findings. In feces, we found for the first time that zinc deficiency induced alterations in the concentrations of nine elements, i.e., increased Mg, Cu, Se, K, Ag, Fe_,_ and Mn, and decreased Zn and Ca.

In sum, we found that zinc deficiency induced alterations in the concentrationsof12 minerals and trace elements in rats, including previously unreported alterations in Ag, As, and Cr (Figs. 3 and 4).

### Reasons for alterations of twelve differential minerals and trace elements

#### Effects of food intake and body weight on the levels of 12 minerals and trace elements

In our study, the food intake of PZG was significantly lower than that of NZG. The intakes of minerals and trace elements in PZG were significantly reduced compared with NZG, and the concentrations of minerals and trace elements in PZG should be reduced. However, the body weights of PZG were significantly lower than the weights of NZG (*P* < 0.05) (Fig. 1). We speculate that decreased body weights may reduce the consumption of minerals and trace elements and maintain their normal levels in the body when the zinc status of rats is normal. Therefore, there were no significant differences in the concentrations of 12 elements in serum, feces, urine, and liver between these two normal zinc diet groups (PZG and NZG).

The PZG group was fed a normal-zinc diet, and their daily food intakes were the same as in LZG. So, the intakes of other minerals and trace elements except for Zinc in PZG were as same as in LZG. The result indicated that food intake had no effect on the concentrations of 12 elements. Compared with PZG, the body weights and feces weights of LZG were significantly decreased. Reduced body and weight feces weights may reduce the consumption and excretion of minerals and trace elements and tried to maintain their level in the body. However, when rats were in a zinc-deficient state, the reduced body weight was not sufficient to maintain the normal levels of the body’s minerals and trace elements. And, the low-zinc diet eventually led to changes in 12 minerals and trace elements. The above results indicated that the alterations of 12 minerals and trace elements in LZG were induced by low zinc diet (or zinc deficiency), and were not related to food intake.

#### Effects of dietary zinc content on the levels of Zn in body

Compared with the normal zinc diet groups (NZG and PZG), for the LZG group, we observed significant decreases in free serum zinc, total zinc in serum, feces, urine, and liver. Therefore, reduced dietary zinc content was the main cause of decreased zinc in the body [25]. To maintain zinc homeostasis, the zinc-deficient rats increased zinc reabsorption, thereby causing decreased concentrations of zinc in feces and urine and decreased excretion via urine and feces (Fig. 4). However, decreased excretion of fecal and urinary zinc was insufficient to meet the zinc demands of serum and liver, so eventually there were decreased concentrations of zinc in these samples.

#### Effect of low-zinc diet on excretion and reabsorption of 10 minerals and trace elements

##### Excretion and reabsorption of mg, cu, se, K, ag, Fe and Mn by feces

The concentrations and excretion of Mg, Cu, Se and K in feces of LZG were all significantly increased compared to NZG, whereas their concentrations in urine and liver were decreased (Fig. 4). These results suggested that zinc deficiency caused increased fecal excretion of these four elements. To maintain the concentrations of these four elements in the body, their urinary excretion was remarkably reduced. But homeostasis still could not be maintained, which led to a further decrease concentration of these four elements in liver. Increased total excretion of Cu and Se suggested that the serum concentrations of Cu and Se may also decline if zinc deficiency was further aggravated.

In contrast, the concentrations of Ag, Fe and Mn in feces were significantly increased in LZG compared with NZG. However, feces excretion of Ag, Fe and Mn were not changed and decreased urinary excretion of Ag and Mn were observed in LZG. So, concentrations of Mn were unchanged in serum, urine and liver. But, lower Mn excretion did not decrease the serum concentration of Mn in LZG. In serum, the concentration of Ag was decreased significantly due to feces excretion, whereas Fe only showed a decreasing trend. And further exacerbation of zinc deficiency may decrease the concentration of serum Fe for higher feces excretion. Brad J. Niles reported that zinc deficiency induced a consequence of alterations in iron regulatory protein-binding activity, iron transporters, and iron storage protein [26].

Increased excretion of Cu, Se and Fe suggested that zinc deficient rats were at risk of Cu, Se and Fe deficiency.

##### Excretion and reabsorption of Na, Cr, and as by urine

Compared with the NZG, the concentrations of Na, As, and Cr in urine of LZG and their excretion were significantly decreased; however, their concentrations were normal in serum, feces, and liver (Figs. 3 and 4). Others have reported that the concentrations of Na and K in cerebellum and hippocampus of zinc-deficient rats were higher than those of normal rats [14]. The decreased K excretion of LZG was also observed in our study. We speculate that the urinary reabsorption of Na and K in zinc-deficient rats was increased to maintain high concentrations of Na and K in other tissues; this resorption, in turn, caused diminished urinary excretion of Na and K. To verify this hypothesis, it is necessary to measure concentrations of Na and K in other tissues.

#### Effect of low-zinc diet on the redistribution of ca

We did not find a significant difference in dietary calcium intake between LZG and PZG (Fig. 1). However, the concentrations of Ca in liver, feces, and urine and excretion of Ca in feces and urine of LZG were remarkably decreased compared with PZG and NZG (Figs. 3 and 4). These results suggested that zinc deficiency increased the reabsorption of calcium by decreasing calcium excretion, so that the calcium of LZG rats was not lost. But where did the decreased calcium go in the LZG rats? In our study, using colorimetry, we did not detect a significant difference in the level of free calcium (Ca^2+^) in serum between LZG and NZG (Table 1). However, the total amount of calcium in LZG serum was significantly increased compared with NZG and PZG (Fig. 2). Total calcium in serum includes free calcium (calcium ion), protein-bound calcium, and other calcium compounds. The absence of a change in free serum calcium suggested that the level of protein-bound calcium or calcium compounds was increased. We speculate that zinc deficiency may increase the distribution of calcium in serum or other tissues and decrease calcium excretion. The possible mechanism is due to the antagonism of zinc and calcium by calcium-activated calmodulin [27, 28]. Zinc is an inhibitor both of calcium-activated calmodulin. Zn deficiency raised the Ca concentration in the brain, muscle, testis by elevated calmodulin concentration [29, 30].

### Adverse effects of alterations in minerals and trace elements

Minerals and trace elements are widely distributed in the body and have important physiological functions. Abnormal availability of minerals and trace elements induces serious adverse effects [19, 31–33].

Decreases in body weight, growth hormone, dietary intake, thymic organ coefficient, IL-1/IL-6, thymosin, and insulin in LZG indicated that zinc deficiency negatively affected growth, immunity, and sugar metabolism in rats (Table 1). It is well known that zinc deficiency is related to growth retardation [34], impaired immunity function [5], and emergence of diabetes [3, 9]. In contrast to zinc deficiency that affects these three aspects of physiology, impaired growth and development is associated with magnesium deficiency [35], selenium, iron, and magnesium deficiencies are related to impaired immune function [32, 36–40], and copper, magnesium, and manganese have important functions in regulation of blood sugar [3, 41]. Therefore, in addition to the direct adverse effects induced by zinc deficiency, changes in other minerals and trace elements induced by zinc deficiency may also indirectly promote the above adverse effects. In addition, iron deficiency can cause anemia [17],copper deficiency affects the occurrence and development of cardiovascular diseases [19, 20], and magnesium and calcium deficiencies are linked to osteoporosis [42]. Recent literature has confirmed that zinc deficiency is closely related to decreased BMD and osteoporosis [43–45]. We hypothesize that the redistribution of calcium in the body induced by zinc deficiency played an important role in decreased BMD and osteoporosis. Therefore, our results suggest that the alterations of minerals and trace elements induced by zinc deficiency may be associated with the above adverse effects.

## Conclusion

Our results confirmed that zinc deficiency induced alterations in the concentrations of 12 minerals and trace elements in serum, urine, feces and liver of rats. These changes occurred by increased excretion of Cu, Se and Fe, decreased excretion of 8 elements, and increased redistribution of calcium in serum or other tissues.

## Supplementary information

Additional file 1: **Table S1.** The main component of rats’ diet for two groups. **Table S2.** The concentrations of 16 elements in serum of rats. **Table S3**. The concentrations of 16 elements in feces of rats. **Table S4.** The concentrations of 16 elements in urine of rats. **Table S5.** The concentrations of 16 elements in liver of rats. **Table S6.**The excretion of 12elements in feces and urine. **Table S7.** The difference of urine and fecal excretion of 12 elements between LZG and NZG. **Figure S1.** Concentrations of three minerals and trace elements in feces between three groups *: *P*<0.05 LZG vs NZG and PZG. **Figure S2.** Concentrations of three minerals and trace elements in urine between three groups *: *P*<0.05 LZG vs NZG and PZG. **Figure S3**. Concentrations of three minerals and trace elements in liver between three groups *: *P*<0.05 LZG vs NZG and PZG. **Figure S4**. Feces weight and Urine volume of Rats *: *P*<0.05, LZG vs NZG and PZG. (DOCX 1270 kb)

## Data Availability

The datasets used and/or analyzed during the current study are available from the corresponding author on reasonable request.

## Abbreviations

AAS: Atomic absorption spectroscopy
BMD: Bone mineral density
CPS: Count per second
ICP-MS: Inductively coupled plasma mass spectrometry
LZG: Low zinc diet group
NZG: Normal zinc diet group
PZG: Pair-fed group
RF: Radiofrequency
