# Supplementary material for: The effects of zinc deficiency on homeostasis of twelve minerals and trace elements in the serum, feces, urine and liver of rats
Source: Nutr Metab (Lond). 2019 Oct 30;16:73. doi: 10.1186/s12986-019-0395-y (PMC6820923; doi:10.1186/s12986-019-0395-y)
Supplement: Supplementary file 1 — Table S1. The main component of rats’ diet for two groups. Table S2. The concentrations of 16 elements in serum of rats. Table S3. The concentrations of 16 elements in feces of rats. Table S4. The concentrations of 16 elements in urine of rats. Table S5. The concentrations of 16 elements in liver of rats. Table S6.The excretion of 12elements in feces and urine. Table S7. The difference of urine and fecal excretion of 12 elements between LZG and NZG. Figure S1. Concentrations of three minerals and trace elements in feces between three groups *: P<0.05 LZG vs NZG and PZG. Figure S2. Concentrations of three minerals and trace elements in urine between three groups *: P<0.05 LZG vs NZG and PZG. Figure S3. Concentrations of three minerals and trace elements in liver between three groups *: P<0.05 LZG vs NZG and PZG. Figure S4. Feces weight and Urine volume of Rats *: P<0.05, LZG vs NZG and PZG. (DOCX 1270 kb) [file 12986_2019_395_MOESM1_ESM.docx]

**Additional file**

**Table S1. The main component of rats’ diet for two groups.**

| **Components** | **Low zinc diet group** | **Normal zinc diet group** |
| --- | --- | --- |
| Energy, kcal/kg | 3766 | 3766 |
| Protein (**Egg white**)calories accounts for, % | 17 | 17 |
| Fat calories account for, % | 14 | 14 |
| Carbohydrate calories account for, % | 69 | 69 |
| protein(**Egg white**), % | 16 | 16 |
| fat, % | 6 | 6 |
| Carbohydrate, % | 66 | 66 |
| **Mineral content (g/kg):** |  |  |
| Calcium carbonate | 357 | 357 |
| Potassium phosphate | 196 | 196 |
| Potassium citrate | 70.78 | 70.78 |
| Potassium sulfate | 46.6 | 46.6 |
| Magnesium oxide | 24 | 24 |
| Sodium chloride | 74 | 74 |
| Cupric carbonate | 0.3 | 0.3 |
| 1%Potassium iodate | 0.01 | 0.01 |
| Ferric citrate | 6.06 | 6.06 |
| Manganous carbonate | 0.63 | 0.63 |
| sodium carbonate | 0.01025 | 0.01025 |
| **Zinc carbonate** | **0** | **1.65** |
| Chromium potassium sulfate | 0.275 | 0.275 |
| ammonium molybdate | 0.00795 | 0.00795 |
| Boric acid | 0.0815 | 0.0815 |
| Sodium fluoride | 0.0635 | 0.0635 |
| Sucrose | 222.676 | 221.026 |
| Total | 998.4942 | 998.4942 |
| **Vitamin content:** |  |  |
| Vitamin A, IU/g | 4.0 | 4.0 |
| Vitamin D, IU/g | 1.0 | 1.0 |
| Vitamin E, IU/kg | 81.6 | 81.6 |
| Vitamin K, ppm | 0.29 | 0.29 |
| Thiamine, ppm | 6.1 | 6.1 |
| Riboflavin, ppm | 6.7 | 6.7 |
| niacin, ppm | 30 | 30 |
| Pantothenic acid, ppm | 16 | 16 |
| Folic acid, ppm | 2.1 | 2.1 |
| Pyridoxine, ppm | 5.8 | 5.8 |
| Biotin, ppm | 0.2 | 0.2 |
| Vitamin B-12, mcg/kg | 29 | 29 |
| Choline chloride, ppm | 1.25 | 1.25 |
| Ascorbic acid, ppm | 0 | 0 |

**Table S2.** **The concentrations of 16 elements in serum of rats**

| **Elements** | **NZG** | **PZG** | **LZG** |
| --- | --- | --- | --- |
| Zn mg/l | 2.11±0.35 | 2.16±0.37 | 1.83±0.10* |
| Ca mg/l | 127.10±11.15 | 129.02±12.37 | 143.26±7.01* |
| Mg mg/l | 47.69±5.07 | 48.34±5.30 | 50.57±2.79 |
| Cu mg/l | 1.68±0.10 | 1.61±0.14 | 1.59±0.18 |
| Na g/l | 3.37±0.15 | 3.33±0.15 | 3.33±0.17 |
| K mg/l | 156.15±12.16 | 157.30±16.82 | 157.18±12.81 |
| Al mg/l | 9.73±1.60 | 9.11±1.02 | 9.28±1.20 |
| Fe mg/l | 22.95±3.34 | 22.90±2.68 | 22.34±2.60 |
| Cr g/l | 10.19±2.63 | 9.01±1.92 | 9.90±2.18 |
| Mn μg/l | 391.18±58.91 | 356.42±42.11 | 372.92±51.38 |
| As μg/l | 21.73±1.37 | 21.84±0.99 | 21.46±0.95 |
| Se μg/l | 448.91±31.12 | 440.53±42.76 | 446.22±38.64 |
| Ag μg/l | 4.84±0.70 | 4.97±0.93 | 3.96±1.02* |
| Ba μg/l | 263.20±39.63 | 264.30±38.43 | 259.26±36.86 |
| Pb μg/l | 58.10±9.24 | 53.25±14.35 | 50.72±14.91 |
| Sn μg/l | 153.03±52.31 | 105.88±94.83 | 177.78±34.26 |

*: *P*＜0.05,LZG vs NZG and PZG.

**Table S3. The concentrations of 16 elements in feces of rats**

| **Elements** | **NZG** | **PZG** | **LZG** |
| --- | --- | --- | --- |
| Zn μg/g | 268.23±38.17 | 268.38±21.38 | 20.66±6.03* |
| Ca mg/g | 10.21±2.68 | 10.12±3.05 | 1.62±1.03* |
| Mg mg/g | 1.76±0.37 | 1.62±0.38 | 3.59±1.04* |
| Cu μg/g | 53.5±7.48 | 52.94±7.45 | 82.00±8.90* |
| Na mg/g | 3.76±0.84 | 3.32±0.89 | 3.37±0.92 |
| K mg/g | 1.85±0.43 | 2.16±0.38 | 3.24±0.73* |
| Al μg/g | 101.70±9.44 | 108.13±6.05 | 100.14±22.51 |
| Fe mg/g | 0.59±0.07 | 0.54±0.14 | 0.87±0.21* |
| Cr mg/g | 55.55±12.36 | 57.92±8.59 | 66.14±16.73 |
| Mn μg/L | 127.69±16.50 | 130.10±11.60 | 170.05±23.98* |
| As μg/g | 0.05±0.06 | 0.07±0.09 | 0.11±0.12 |
| Se mg/g | 0.91±0.13 | 0.90±0.15 | 1.35±0.26* |
| Ag μg/g | 0.02±0.01 | 0.03±0.01 | 0.04±0.02* |
| Ba μg/g | 4.47±0.57 | 4.81±1.03 | 4.5±0.82 |
| Pb μg/g | 0.89±0.35 | 0.70±0.17 | 0.91±0.38 |
| Sn μg/g | 0.46±0.19 | 0.63±0.34 | 0.63±0.28 |

*: *P*＜0.05,LZG vs NZG and PZG.

**Table S4. The concentrations of 16 elements in urine of rats**

| **Elements** | **NZG** | **PZG** | **LZG** |
| --- | --- | --- | --- |
| Zn mg/l | 8.11±2.53 | 9.44±2.69 | 4.28±1.09* |
| Ca g/l | 0.94±0.37 | 0.92±0.32 | 0.33±0.21* |
| Mg mg/l | 422.21±144.08 | 488.80±157.73 | 124.32±66.43* |
| Cu mg/l | 1.66±0.17 | 1.77±0.20 | 1.50±0.17* |
| Na g/l | 3.11±1.02 | 2.92±1.10 | 1.66±0.43* |
| K g/l | 3.96±1.08 | 4.18±0.52 | 3.13±0.74* |
| Al mg/l | 10.90±2.21 | 10.73±2.63 | 10.35±1.68 |
| Fe mg/l | 29.63±4.46 | 29.51±4.62 | 27.79±6.39 |
| Cr g/l | 3.44±0.49 | 3.48±0.49 | 3.06±0.35* |
| Mn μg/l | 593.38±139.31 | 588.25±158.26 | 506.09±166.77 |
| As μg/l | 67.41±5.04 | 67.93±7.37 | 57.30±9.03* |
| Se μg/l | 342.78±72.56 | 306.38±117.69 | 250.47±69.75* |
| Ag μg/l | 5.97±1.18 | 5.91±1.39 | 5.94±0.84 |
| Ba μg/l | 321.58±52.58 | 304.96±56.97 | 298.01±50.3 |
| Pb μg/l | 130.13±18.91 | 130.05±12.07 | 125.69±22.57 |
| Sn μg/l | 25.03±5.31 | 25.42±4.63 | 25.78±6.34 |

*: *P*＜0.05,LZG vs NZG and PZG.

**Table S5. The concentrations of 16 elements in liver of rats**

| **Elements** | **NZG** | **LZG** |
| --- | --- | --- |
| Zn μg/g | 60.22±5.46 | 47.26±2.89* |
| Ca μg/g | 133.00±10.41 | 22.11±6.74* |
| Mg μg/g | 307.92±11.43 | 274.29±26.99* |
| Cu μg/g | 9.44±0.60 | 7.62±1.02* |
| Na μg/g | 776.67±19.82 | 802.14±107.00 |
| K mg/g | 4.01±0.24 | 3.44±0.42* |
| Al μg/g | 21.00±3.84 | 19.42±2.85 |
| Fe μg/g | 334.08±62.76 | 367.50±56.01 |
| Cr μg/g | 8.30±0.77 | 7.69±0.28 |
| Mn μg/g | 4.06±0.24 | 4.05±0.39 |
| As μg/g | 0.53±0.50 | 0.88±0.57 |
| Se μg/g | 1.29±0.13 | 0.83±0.13* |
| Ag ng/g | 12.67±1.54 | 12.86±2.67 |
| Ba ng/g | 482.33±109.68 | 398.33±26.39 |
| Pb μg/g | 5.64±3.09 | 6.03±3.38 |
| Sn μg/g | 0.32±0.21 | 0.33±0.13 |

*: *P*＜0.05,LZG vs NZG.

**Table S6.The excretion of 12elements in feces and urine**

|  | **Feces** | | |  | **Urine** | | | |
| --- | --- | --- | --- | --- | --- | --- | --- | --- |
|  | **NZG** | **PZG** | **LZG** |  | **NZG** | **PZG** | **LZG** | |
| Zn (μg) | 502.66±71.52 | 481.47±38.353 | 29.52±8.62*^#^ |  | 70.72±23.66 | 70.09±19.94 | | 32.93±8.38*^#^ |
| Ca (mg) | 19.13±5.02 | 18.151±5.474 | 2.31±1.48*^#^ |  | 8.25±3.41 | 6.83±2.36 | | 2.52±1.58*^#^ |
| Mg (mg) | 3.29±0.7 | 2.907±0.689 | 4.6±2.14*^#^ |  | 3.77±1.26 | 3.63±1.17 | | 0.92±0.51*^#^ |
| Cu(μg) | 100.26±14.02 | 94.97±13.363 | 117.18±12.71*^#^ |  | 14.41±1.61 | 13.15±1.47 | | 11.49±1.32*^#^ |
| Se(μg) | 1705.89±245.36 | 1619.743±261.03 | 1934.5±366.04^#^ |  | 2.97±0.63 | 2.66±0.87 | | 1.92±0.53*^#^ |
| K (mg) | 3.46±0.8 | 3.882±0.68 | 4.64±1.05* |  | 35±9.65 | 31.01±3.84 | | 23.99±5.63*^#^ |
| Na (mg) | 7.05±1.58 | 6.185±1.602 | 4.82±1.31*^#^ |  | 27.85±8.72 | 21.69±8.17 | | 12.74±3.33*^#^ |
| Cr (mg) | 104.1±23.15 | 103.902±15.403 | 94.52±23.9 |  | 29.93±4.37 | 25.87±3.63* | | 23.56±2.66*^#^ |
| As (ng) | 185.71±70.81 | 258.755±128.339 | 221.74±163.24 |  | 583.97±46.04 | 504.22±54.73 | | 436.13±69.15*^#^ |
| Ag (ng) | 46.28±12.92 | 59.561±10.961 | 51.17±20.72 |  | 51.24±10.83 | 43.9±10.29 | | 45.6±6.46* |
| Fe(μg) | 1004.42±125.82 | 967.971±242.915 | 1250.15±305.5*^#^ |  | 252.19±37.31 | 219.05±34.29* | | 215.43±48.95* |
| Mn(μg) | 239.3±30.92 | 233.398±20.808 | 230.85±63.76 |  | 4.97±1.1 | 4.37±1.17 | | 3.92±1.28* |

*: *P*＜0.05,LZG vs NZG ;#: *P*＜0.05,LZG vs PZG.

**Table S7. The difference of urine and fecal excretion of 12 elements between LZG and NZG**

|  | **Fecal excretion**  (LZG-NZG) | **Urine excretion**  **(**LZG-NZG**)** | **Total excretion**  (Fecal excretion+ Urine excretion) |
| --- | --- | --- | --- |
| Zn(μg) | -473.14 | -37.79 | -510.93 |
| Ca(mg) | -16.82 | -5.73 | -22.55 |
| Mg(mg) | 1.31 | -2.85 | -1.54 |
| Cu(μg) | 16.92 | -2.92 | 14 |
| Se(μg) | 228.61 | -1.05 | 227.56 |
| K(mg) | 1.18 | -11.01 | -9.83 |
| Na(mg) | -2.23 | -15.11 | -17.34 |
| Cr(mg) | -9.58 | -6.37 | -15.95 |
| As(ng) | 36.03 | -147.84 | -111.81 |
| Ag(ng) | 4.89 | -5.64 | -0.75 |
| Fe (μg) | 145.73 | -36.76 | 108.97 |
| Mn (μg) | -8.45 | -1.05 | -9.5 |


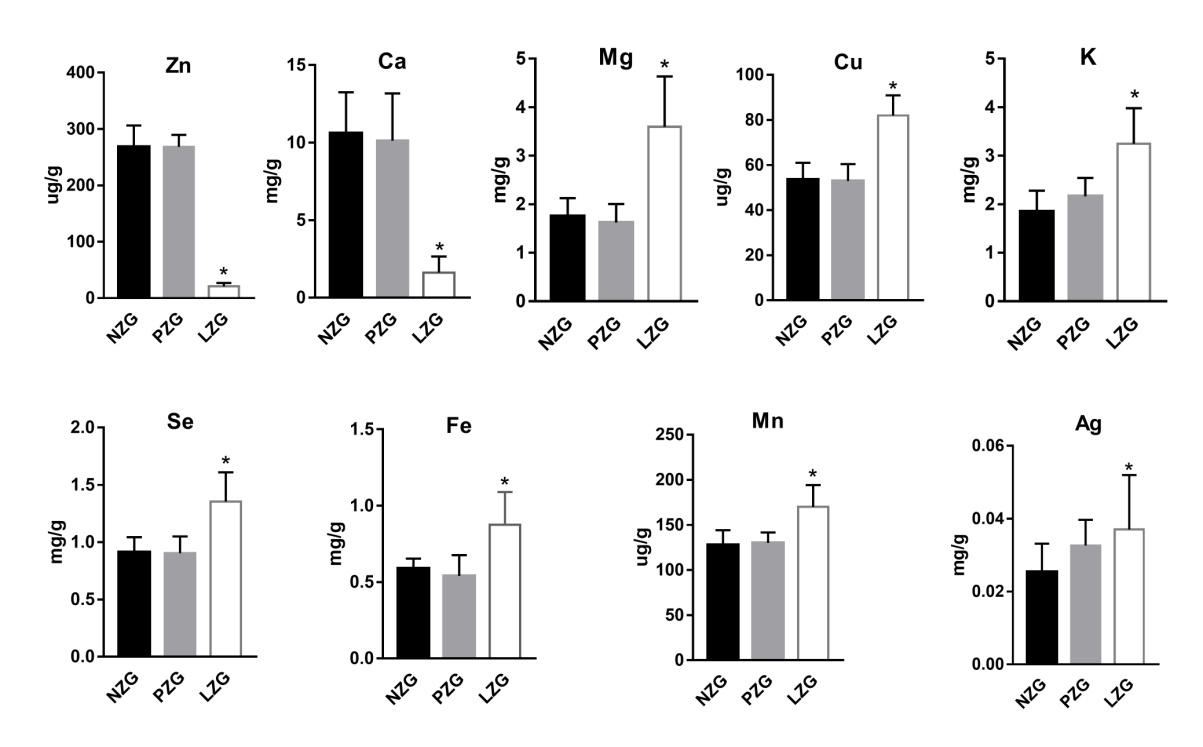


**Figure S1 Concentrations of three minerals and trace elements in feces between three groups** *: *P*＜0.05 LZG vs NZG and PZG.


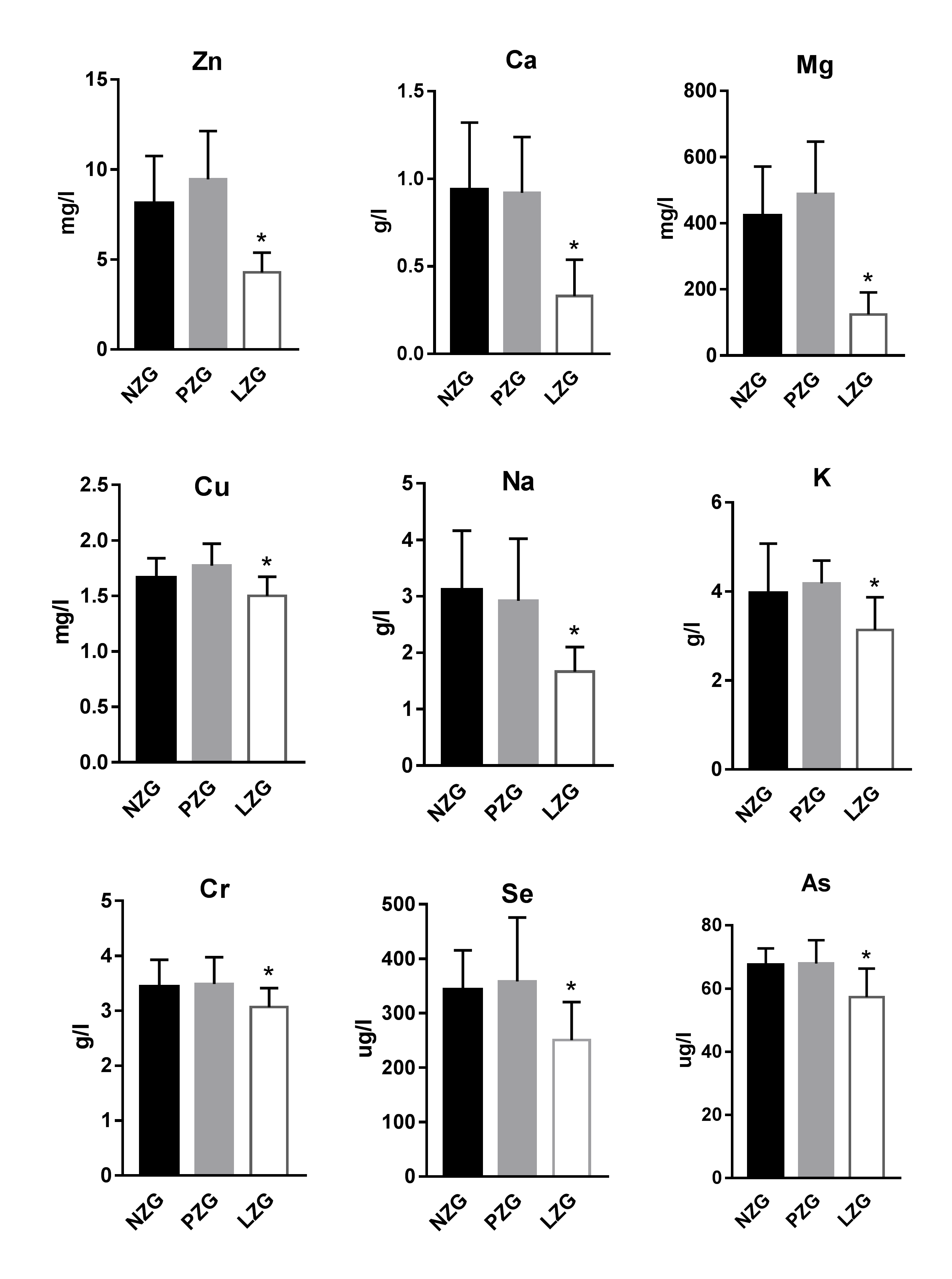


**Figure S2 Concentrations of three minerals and trace elements in urine between three groups** *: *P*＜0.05 LZG vs NZG and PZG.


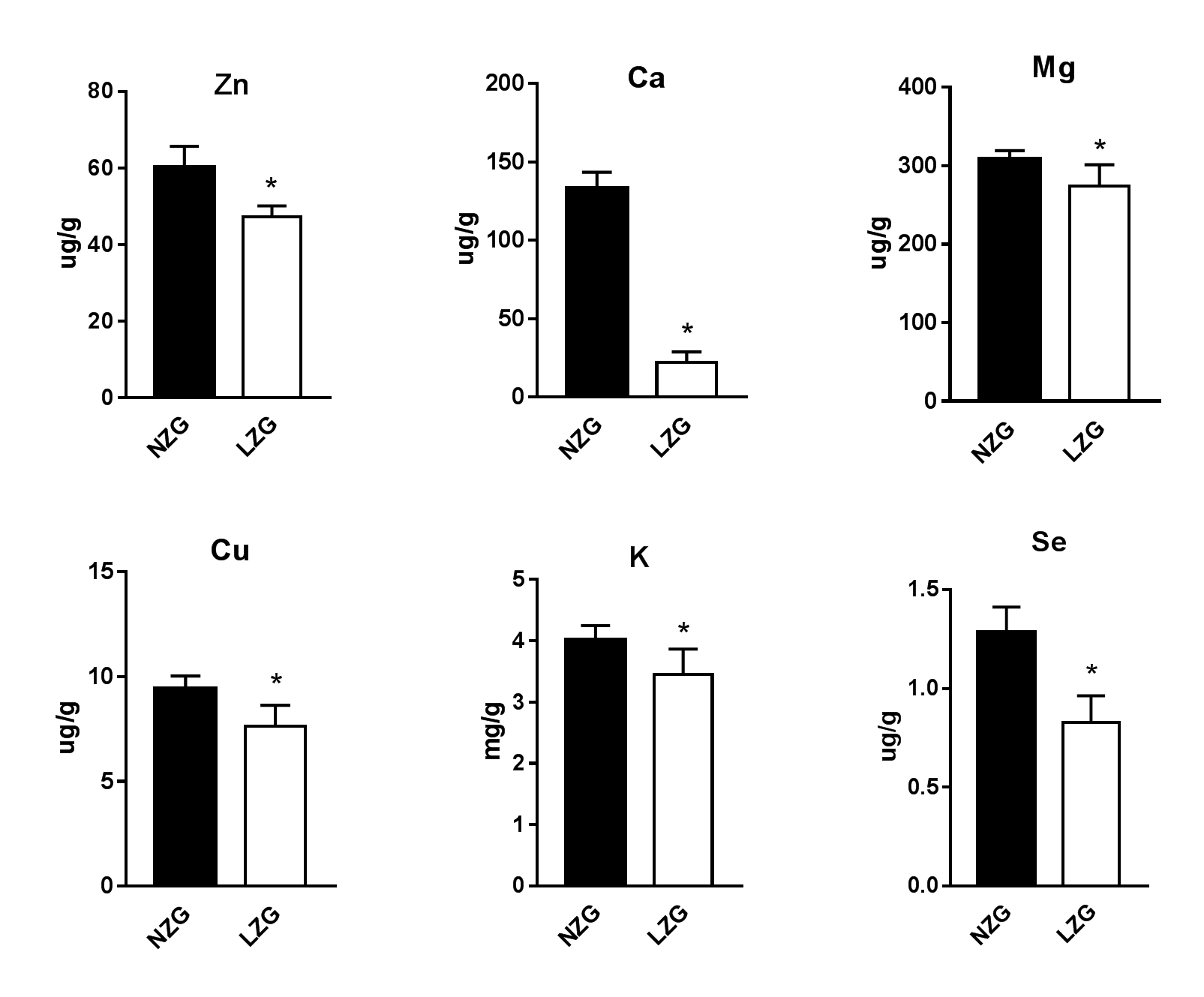


**Figure S3 Concentrations of three minerals and trace elements in liver between three groups** *: *P*＜0.05 LZG vs NZG and PZG.

**
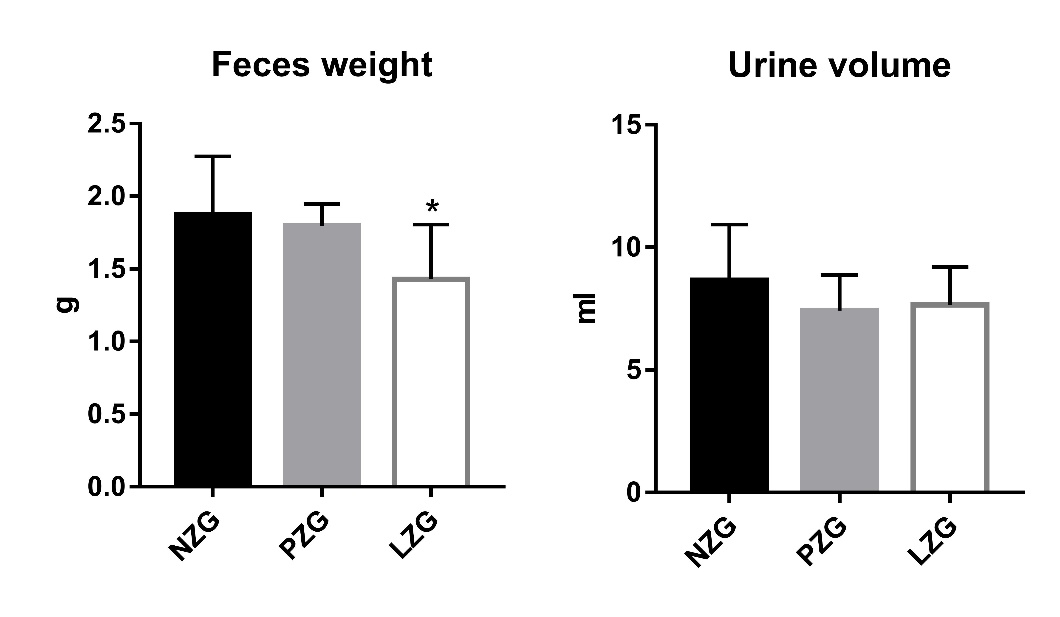
**

**Figure S4. Feces weight and Urine volume of Rats**

*: *P*＜0.05, LZG vs NZG and PZG.
